# The “Usual Suspects”: Genes for Inflammation, Fibrosis, Regeneration, and Muscle Strength Modify Duchenne Muscular Dystrophy

**DOI:** 10.3390/jcm8050649

**Published:** 2019-05-10

**Authors:** Luca Bello, Elena Pegoraro

**Affiliations:** Department of Neurosciences, University of Padova, 35128 Padova, Italy; elena.pegoraro@unipd.it

**Keywords:** Duchenne muscular dystrophy, genetic modifiers, osteopontin, *SPP1*, *LTBP4*, *CD40*, *ACTN3*, *THBS1*

## Abstract

Duchenne muscular dystrophy (DMD), the most severe form of dystrophinopathy, is quite homogeneous with regards to its causative biochemical defect, i.e., complete dystrophin deficiency, but not so much with regards to its phenotype. For instance, muscle weakness progresses to the loss of independent ambulation at a variable age, starting from before 10 years, to even after 16 years (with glucocorticoid treatment). Identifying the bases of such variability is relevant for patient counseling, prognosis, stratification in trials, and identification of therapeutic targets. To date, variants in five loci have been associated with variability in human DMD sub-phenotypes: *SPP1*, *LTBP4*, *CD40*, *ACTN3*, and *THBS1*. Four of these genes (*SPP1*, *LTBP4*, *CD40*, and *THBS1*) are implicated in several interconnected molecular pathways regulating inflammatory response to muscle damage, regeneration, and fibrosis; while *ACTN3* is known as “the gene for speed”, as it contains a common truncating polymorphism (18% of the general population), which reduces muscle power and sprint performance. Studies leading to the identification of these modifiers were mostly based on a “candidate gene” approach, hence the identification of modifiers in “usual suspect” pathways, which are already known to modify muscle in disease or health. Unbiased approaches that are based on genome mapping have so far been applied only initially, but they will probably represent the focus of future developments in this field, and will hopefully identify novel, “unsuspected” therapeutic targets. In this article, we summarize the state of the art of modifier loci of human dystrophin deficiency, and attempt to assess their relevance and implications on both clinical management and translational research.

## 1. Introduction

This review aims at providing a state of the art regarding genetic loci that are associated with a modifier effect on the phenotype of human Duchenne muscular dystrophy (DMD, MIM #310200). This disease is part of a group of allelic disorders, called dystrophinopathies, whose phenotypic spectrum is vast. Therefore, in order to understand DMD modifiers, we should start by providing a precise definition of DMD—a task that, despite the immense *corpus* of scientific literature regarding DMD, is less simple and straightforward than it may appear.

DMD is a lethal, X-linked neuromuscular disorder with a pediatric onset, which affects around 1 in 5000–10,000 male live births [1]. Typically, DMD is characterized by proximal muscle weakness presenting in toddlers or young boys, which progresses to the loss of independent ambulation (LoA) by the teenage years, and is followed by the loss of upper limb function. In subsequent years, restrictive respiratory insufficiency and dilated cardiomyopathy (DCM) significantly reduce life expectancy [2].

The cause of DMD are mutations in the *DMD* gene (MIM *300377) that disrupt the open reading frame (ORF) of the transcript, leading to the absence of full-length dystrophin protein at the plasma membrane of skeletal muscle fibers and cardiomyocytes [3]. Conversely, mutations that respect the ORF lead to the expression of qualitatively and/or quantitatively altered dystrophin, result in the milder allelic form of dystrophinopathy, Becker muscular dystrophy (BMD, MIM #300376) [4]. BMD has a lower incidence of about 1 in 30,000 males [5], a later onset, and a slower, more variable progression [6,7,8].

The classification of dystrophinopathic patients into DMD vs. BMD is somewhat blurred, and it relies on three orders of evaluations: the characterization of *DMD* mutations at the genomic and transcriptomic level (i.e., predicted disruption of the ORF in DMD vs. preservation in BMD); protein assays performed on bioptic muscle tissue samples, including immunohistochemistry (IHC) and immunoblot, which show completely absent or trace amounts of dystrophin in DMD, as opposed to reduced but present dystrophin, usually with abnormal molecular weight, in BMD; and, clinical criteria, taking into consideration that the presence of weakness by the age of five years, and/or LoA by the age of 13 years are typical for a DMD phenotype, while LoA beyond 16 years is suggestive of BMD. The latter (clinical) criterion is challenged by the improved clinical course of DMD after the implementation of modern rehabilitation protocols and glucocorticoid corticosteroid (GC) treatment, which have a shifted median age at LoA from around 10 to around 14 years [9]. Cases with borderline or discordant genetic and protein assays, and an intermediate clinical picture, are sometimes labeled “intermediate dystrophinopathy” (IMD). Probably because DMD vs. BMD may be distinguished on three different axes—genetic, proteic, and clinical—which are often, but not always, concordant, it does not come as a surprise that a univocal, universally shared definition of the exact boundaries between the two conditions has never been reached.

In fact, while dystrophinopathies are traditionally dicotomized into well-defined DMD and BMD categories, their phenotypic spectrum may also be seen as continuous, spanning from severe to mild: severe DMD cases with delayed motor milestones (although never with a congenital onset) and LoA as early as seven years of age; “typical” DMD; IMD; “typical” BMD, in which an overt weakness of the pelvic girdle and thighs is present and LoA ensues at variable ages during adult life; “mild” BMD in which weakness is less dramatic, being often confined to thigh muscles, and there is no LoA; and, finally, virtually asymptomatic cases, with preserved muscle strength and function, even in elderly patients. A full description of the whole phenotypic spectrum of dystrophinopathies in affected males, not to mention manifesting female carriers, is beyond the scope of this review. Here, as stated above, we will focus on the severe end of the dystrophinopathy spectrum, i.e., DMD, synthetically defined as severe dystrophinopathy with absent, or at most trace amounts of dystrophin protein expression; and we will aim to summarize current knowledge regarding the molecular bases of DMD phenotypic variability.

## 2. Phenotype Variability in DMD

Within the boundaries of the clinical/molecular definition of DMD, phenotype variability is relevant [10]. Age at LoA is primarily used to measure this variability, because of the impact of LoA on daily life and the overall functioning of patients, and its correlation with other disease milestones, such as the onset of respiratory insufficiency, the need for scoliosis surgery, and overall survival [11]. Even with the implementation of GC treatment and the current standards of care, LoA may occur as early as the age of seven or eight in the most severe cases, while at the opposite end of the DMD spectrum, LoA may be delayed up to the age of 16 (without GC treament) or even beyond 20 (with GCs) [12], despite out-of-frame *DMD* mutations and absent dystrophin, as assayed by IHC and immunoblot. The onset and rate of progression of respiratory insufficiency [10] and DCM are also variable by several years [13], sometimes even by decades (e.g., DMD patient in their forties with no detectable DCM).

The main scientific question regarding DMD genetic modifiers may be summarized as such: if two DMD patients both completely lack dystrophin, what mechanisms may cause different levels of phenotypic severity between them?

Before attempting to answer, it should be stressed that, even within the patient population that is usually classified as DMD, dystrophin may actually not always be completely absent from skeletal muscle fibers. Protein assays that are commonly used in the diagnostic setting (IHC and immunoblot) have limited sensitivity, so that small amounts of protein may escape detection, while still exerting a measurable effect on the phenotype. These trace protein amounts most often derive from exceptions to the reading frame rule. Examples of such exceptions are: translational reinitiation downstream of frameshift mutations in the N-terminal domain of dystrophin [14,15,16,17,18], the splicing-out of in-frame exons of the rod domain containing a nonsense mutation [19], and alternative splicing of exons adjacent to the deletion boundaries, resulting in a restored ORF [20]. It is now well established that these cases of low-level rescue of dystrophin expression are associated with milder DMD or IMD phenotypes, as is the case for the deletion of exons 3 to 7 [16,17,18,21] and the isolated deletion of exon 45 [18,22,23]. Therefore, when comparing disease severity among DMD patients, not only environmental factors, such as standards of care and socioeconomic status [24], should be taken into consideration, but also the fact that some frameshift mutations are more likely to be associated to trace-level dystrophin expression. This should not be confused with the phenomenon of revertant fibers, which is probably due to a restoration of the ORF at the genomic level with a cell-autonomous mechanism, in fibers that are derived from a clonal population of satellite cells, probably because of epigenetic events altering splicing. Additionally, the fact that the quantity and the extension of revertant fibers correlates more with age at muscle biopsy than with overall phenotype severity is suggestive of a somatic event [25,26]. While revertant fibers are grouped in small, isolated clusters in the context of an otherwise dystrophin-negative muscle sample, dystrophin traces are diffused at a low, but fairly homogeneous, level throughout the sample.

Once considered the effects of unequal, or evolving standards of care (environmental factors), and of certain *DMD* mutations (“cis-acting” genetic modifiers), the answer to the question of phenotype variability in dystrophin-null DMD patients lies in their genetic background. In other words, among the myriad variants that are contained in the human genome, some may epistatically interact with the disease-causing mutation, modulating its phenotype. These “trans-active” genetic modifiers are the main focus of the present review.

The identification of genetic modifiers of a severe, so far incurable, disease like DMD is relevant for several reasons. First and foremost, the modifiers may represent novel therapeutic targets. In fact, if a variant in a gene is capable of modifying the DMD phenotype by altering its protein product or level of expression, a drug directed to this target may obtain the effect of ameliorating DMD pathology. Moreover, an improved understanding of the wide variation that is observed in the trajectories of disease progression among patients would lead to better design of clinical trials, as it would allow for the selection or stratification of recruited patients according to genotype, or improve the interpretation of results by implementing genotypes as covariates in post-hoc analyses. The use of modifier genotype for prognostic predictions in individual patients appears to be less realistic, as the expected size of modifier effects may be relatively small in relation to many other variables, and that the interaction of different modifier genotypes in the same individual adds complexity to the prediction.

The potential strategies for the identification of genetic modifiers of a Mendelian disease are basically of two types: hypothesis-driven, i.e., candidate gene association studies, and hypothesis-free, i.e., genome-wide association studies (GWAS). The former studies concentrate on selected variants in genes with a known role in the pathogenesis of the disease, established in previous preclinical and tissue-based studies. The advantage of this design is that it poses a focused scientific question, which requires relatively small sample sizes in order to be answered with sufficient statistical power. Most of the modifiers identified so far belong to this category, hence the “usual suspects” in the title of this review. On the other hand, only GWAS has the potential for identifying novel, unsuspected modifier genes, and therefore novel disease pathways. However, because of a massive number of parallel statistical tests of genotype/phenotype association, and the subsequent stringent type I error rate, large sample population sizes are required in GWAS. These sample sizes are very challenging, although not altogether impossible, to collect in rare diseases such as DMD.

## 3. *SPP1* (Secreted PhosphoProtein 1, Also Known as Osteopontin)

The seminal work in the field of DMD genetic modifiers, which was carried out by our group at the University of Padova in collaboration with the Cooperative International Neuromuscular Research Group (CINRG), was a candidate gene study, although the identification of the successful candidate originally derived from a genome-wide approach, i.e., a gene expression array. The gene expression profiles were compared between a few bioptic muscle samples that were obtained from DMD patients who would, in the years subsequent to the biopsy, experience poor response to GC treatment and early LoA, vs. samples from DMD patients with an excellent response to treatment and late LoA. Several differentially expressed genes were identified, and the literature was searched for “functional” single nucleotide polymorphisms (SNPs) within these genes, i.e., SNPs that were associated with some effect on protein expression or health/disease phenotypes. The DMD cohorts from Padova (*n* = 106) and the CINRG Duchenne Natural History Study (DNHS, *n* = 156) were genotyped, and the SNPs were associated with several phenotypes: age at loss of ambulation and a composite score of manual muscle testing scores in the Padova cohort, and grip strength and quantitative muscle testing in the CINRG cohort. Among the differentially expressed genes, *SPP1* resulted to be significantly overexpressed in muscle samples from patients with severe DMD. The SNP rs28357094, situated in the promoter (five bases upstream of the transcription start site) of the *SPP1* gene (MIM *166490), enconding osteopontin, was predicted to alter transcriptional efficiency. Therefore, rs28357094 was genotyped in the Padova cohort, and the effect size of the polymorphism was estimated as approximately one-year earlier LoA in patients carrying one or two copies (dominant model) of the minor G allele at rs28357094. rs28357094 was then validated in the DNHS cohort using different muscle phenotypes (grip strength), confirming the association of the rare G allele with greater weakness. The minor allele frequency (MAF) of this SNP in populations of European ancestry is around 24%, meaning that a considerable proportion of DMD patients is at risk of earlier LoA because of this genotype. The report of these findings, which was published in 2011 [27], was accompanied by an editorial [28], which highlighted the importance of genetic modifier studies for predicting the severity of DMD, while reminding that further validation in independent cohorts is crucial, in order to confirm this kind of genetic associations, extend them to the general DMD population, and eventually use them for clinical trial design and prognosis.

Osteopontin is a versatile cytokine that belongs to the family of small integrin-binding ligand N-linked glycoprotein (SIBLING) secreted phosphoproteins, originally described in bone—hence the name—but later found to be expressed in several organs, especially in response to tissue damage, and with important roles in tissue repair and regeneration, regulation of the inflammatory response, and tumor progression and metastasis [29,30,31]. Osteopontin is subject to alternative splicing and heavy post-translational modifications, including cleavage by thrombin, phosphorylation, and transglutaminase-mediated polymerization [32]. The complexity of these events in different stages of several biological processes poses a challenge to mechanistic studies of the function of this cytokine.

**Figure 1:**
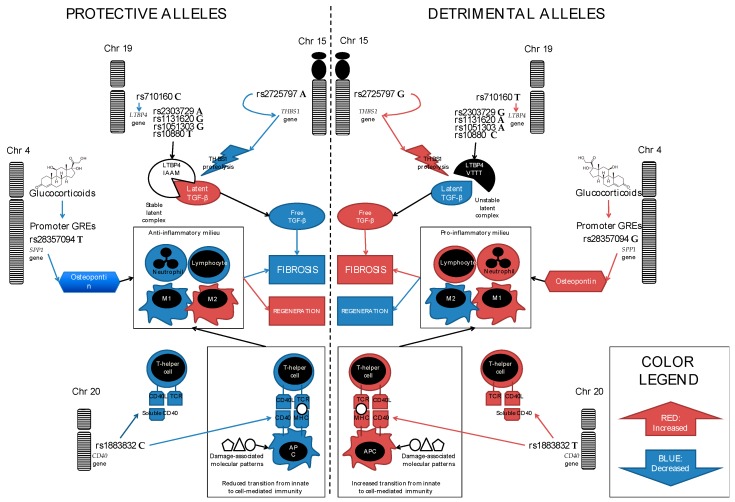
Diagram of the molecular mechanisms proposed to explain the associations of modifier single nucleotide polymorphisms (SNPs) with Duchenne muscular dystrophy (DMD) phenotypes. The color blue indicates molecules, pathways, or biological processes that are reduced in association with a certain genotype; while the color red indicates those that are increased. On the left hand side are represented protective alleles of the modifier SNPs, and their consequences; while the right hand side specularly represents detrimental alleles. Note that only genes with mechanisms related to fibrosis and inflammation are represented here. *ACTN3*, which modifies DMD through different mechanisms related to the sarcomere and muscle fiber type, is excluded.

Osteopontin, even before the identification of the rs28357094 modifier effect, had already been the object of attention from those interested in muscle pathology. Since the early 2000s, the expression profiles of cardiotoxin-damaged murine muscles had shown that *SPP1* transcription is dramatically upregulated 48 h after damage, and osteopontin was localized by IHC around infiltrating macrophages, or necrotic fibers that are engulfed by macrophages, which suggests that, through its integrin-binding adhesive role, osteopontin guides macrophage invasion and the remodeling of damaged muscle tissue [33]. Osteopontin had also been studied in the *mdx* murine model of DMD, specifically by crossing *mdx* with osteopontin-null mice [34]. The resulting double-mutant mouse (DMM) showed a reduction in the severity of fibrosis, increased strength, and reduced amounts of transforming growth factor β (TGFβ), which is a well-known driver of fibrosis in the later phases of DMD pathology [35]. Furthermore, this model exhibited different infiltrating immune cell populations, with reduced natural-killer T cells and neutrophils, increased regulatory T cells, and a modified polarization of macrophages toward the pro-regenerative M2, and away from the pro-inflammatory M1 phenotype. These modifications of of the inflammatory cell pool (Figure 1) may increase the efficiency of regeneration, and delay end-stage fibrosis [36]. Summarizing the findings on osteopontin in animal models of muscle disease, it appears that muscle damage strongly induces osteopontin, probably through nuclear factor κB (NF-κB) responsive elements in the promoter; that its absence delays tissue repair in models of acute damage, but conversely reduces fibrosis due to the chronic damage that is caused by dystrophin deficiency; and that, ultimately, it is involved in different phases of muscle remodeling with various, sometimes contrasting, pro-inflammatory and pro-regenerative effects [37].

Later, gene expression studies regarding human muscular dystrophy samples, including dystrophinopathies and congenital muscular dystrophies, confirmed that, among several genes that are involved in fibrosis and TGFβ signaling, *SPP1* was one of the more constantly and dramatically upregulated, which suggests a key role in dystropathology [38]. Further studies of acute muscle damage, which were conducted through muscle self-reimplantation in normal and osteopontin-null mice, compounded the relevant role of osteopontin in muscle damage and repair, as both inflammatory/necrotic phenomena and the setting of regeneration are delayed in the absence of osteopontin [39]. Other than macrophages, regenerating myoblasts themselves also secrete osteopontin during regeneration, which supports its contribution to the modifications of the extracellular matrix that accompany the formation of nascent myotubes and new myofibers [40]. 

This complex picture of the role of osteopontin in muscle pathology provides a background for the interpretation of *SPP1* genotype associations with human DMD. The G allele at rs28357094 had been previously demonstrated, through electrophoretic mobility shift assays, to determine the reduced binding of the ubiquitous transcription factor Sp1, and subsequently reduce *SPP1* transcription, as assayed by a luciferase reporter downstream of the *SPP1* promoter [41]. A corollarium to these findings, if they were to be translated to human dystrophin-deficient muscle, would be that DMD patients carrying the G genotype (those who lost ambulation earlier in the original report of *SPP1* as a DMD modifier [27]) are expected to express less osteopontin. This hypothesis is in contrast with the findings in the DMM model described above, in which the absence of both dystrophin and osteopontin brought about a milder phenotype. Our group studied osteopontin expression levels in human DMD and control muscle tissue samples in order to investigate this problem [42]. While a strong upregulation of *SPP1* was observed in DMD when compared to normal muscle, as expected and previously reported, no significant difference was found at the mRNA or protein level between DMD patients with the TT vs. TG/GG genotype at rs28357094, which raised doubts regarding the actual mechanistic effects of the SNP. On the other hand, TG/GG muscles did show a reduced amount of CD68+ (macrophage) infiltrating cells, again highlighting the importance of osteopontin in regulating the inflammatory milieu. These intriguing, but perplexing, findings pointed away from the obvious model of a transcriptional effect of the rs28357094 promoter SNP, and suggested that additional complex factors might be at play.

Meanwhile, the DMD research community was at work to provide an independent validation of the *SPP1* modifier effect. The Italian DMD Network provided evidence that, in a longitudinally evaluated cohort of DMD patients (*n* = 80), those carrying the dominant G genotype, after one year of follow-up, experienced a faster deterioration of some ambulation-related outcome measures: the six-minute walk test (6MWT) and the North Star Ambulatory Assessment (NSAA) [43]. Other than providing an independent validation of the detrimental effect of the rs28357094 G allele on DMD progression, these findings provided a proof of concept that trans-active variants in non-disease-causing genes may be relevant for the design and interpretation of clinical trials for rare diseases, as the time of observation and outcomes were the same as those that are commonly adopted in trials. However, shortly after, further validation studies of the effect of rs28357094 on age at LoA gave contrasting results, as no effect was demonstrated in the DMD cohorts collected by the multi-centric European BioNMD consortium (*n* = 336) [44] and in the severe dystrophinopathy cohort studied by the United Dystrophinopathy Project (UDP) group in the United States (*n* = 254) [45]. Conversely, the LoA-delaying effect was confirmed in the CINRG-DNHS cohort, which had been expanded (*n* = 283) and then followed up for a longer time since the original report, with a similar effect size when compared to the previous report in the Padova cohort (one-year difference in median LoA) [46]. In the latter study, the SNP effects were analyzed in stratified sub-populations, according to whether participants had been exposed to at least one-year of GC treatment before LoA or last follow-up. Strikingly, the GC-treated subpopulation exhibited a larger rs28357094 effect, with two-year earlier LoA in patients with the dominant G genotype, while, in the GC-untreated subpopulation, the median age at LoA was identical between the genotypes.

This observation suggested the possibility of an interaction between *SPP1* genotype and glucocorticoids, so that the *SPP1* genotype becomes more relevant in GC-treated than untreated DMD populations. This could represent one possible explanation of the negative findings in the UDP and Bio-NMD cohorts, which had a relatively low proportion of GC-treated participants (about 54% and 30%, respectively). On the other hand, it has to be considered that the effect size of the SNP was not very large, in absolute terms, even in the discovery and the positive validation cohorts (although comparable with the efficacy of currently available treatments), and many other confounders may be involved, such as ethnicity and environmental factors. The hypothesis of an interaction between *SPP1* and GCs has ground in *in vitro* studies of the *SPP1* promoter activity based on mutagenesis and in-gel shift assays [47]. These showed that the stretch of DNA sequence containing the rs28357094 SNP is not only needed in tumor cell lines to maintain high levels of *SPP1* expression, probably through Sp1 transcription factor binding, but it also includes a non-palindromic glucocorticoid receptor responsive element (GRE). Other (palindromic) GREs, as well as estrogen responsive elements (EREs), are predicted by DNA sequence analysis several hundreds of bases more proximally in the promoter sequence. Palindromic consensus sites for steroid receptors bind dimerized receptors, and they usually act as transcriptional enhancers (trans-activators), while non-palindromic GREs/EREs bind monomer receptors, and they are more often trans-repressors, although the net transcriptional effect of any element ultimately depends on the genomic, epigenomic, and hormonal context [48]. Anyhow, the presence of GREs and EREs in the *SPP1* promoter suggests a complex regulation of the *SPP1* promoter by steroid hormones, as is not uncommon for genes that are involved in inflammation. A more recent study of osteopontin in murine and human muscle physiology [49] confirmed, on the one hand, that *SPP1* expression rapidly increases and decreases in the few days after acute muscle damage in mice; on the other hand, in young healthy human female subjects, muscle size was increased by 17% in association with the G allele. The authors hypothesized a sexually dimorphic effect of osteopontin on muscle remodeling, which possibly depends on estrogen-mediated regulation of the *SPP1* promoter. Furthermore, there were tentative associations between the G allele and biomarkers of muscle damage (higher serum CK and myoglobin after a bout of maximal eccentric exercise). The latter finding was validated in a subsequent study on young healthy volunteers [50], in which the female participants carrying the dominant G genotype reached higher levels of serum CK, and showed markedly more signs of myoedema/inflammation in muscle magnetic resonance imaging. This study also included *in vitro* measurements of expression levels of a luciferase reporter downstream of the *SPP1* promoter sequence, before and after treatment with estrogens, which demonstrated an increase in expression after only a T>G substitution at the rs28357094 position by targeted mutagenesis. Supposedly, the reduced binding of the Sp1 transcription factor to the DNA sequence containing the G nucleotide renders the promoter less functional at the baseline, but more responsive to the ERE enhancer effects. A similar mechanism, which involved GREs instead of EREs, may be postulated in DMD patients who take GCs (Figure 1). With the G genotype, resulting in reduced Sp1 binding and dysregulated baseline expression levels, a GRE enhancer effect on *SPP1* expression may lead to increased levels of osteopontin in dystrophic muscle, and in turn to increased chemotaxis of neutrophils, and a tilt of the balance between the M1 and M2 macrophages towards the pro-inflammatory, pro-fibrotic M1 subtype.

In order to investigate the hypothesis that GCs enhance osteopontin expression in the G genotype, the *SPP1* transcript and osteopontin protein expression levels were studied in primary myoblasts that were cultured from DMD and control muscle biopsies, and deriving myotubes, before and after treatment with increasing concentrations of deflazacort [51]. Osteopontin was detectable by immunoblot as a doublet of two bands at 55 and 50 kDa in myoblasts myotubes, respectively. The shift towards the lighter isoform after differentiation was an interesting finding. Also importantly, the administration of deflazacort did, as hypothesized, increase both the *SPP1* transcript and protein in dystrophin-deficient myotubes carrying the rs28357094 G allele (dominant model). A multivariate analysis confirmed a significant interaction between the G allele and deflazacort treatment. These findings strengthened the model, according to which *SPP1* overexpression causes a worse motor outcome in GC-treated, but not in untreated DMD patients. However, the size of this effect is not sufficient to recommend withdrawal from GCs of patients carrying the G genotype, or even a reduction of the dose. In fact, as explained above, modifier genotypes are better applied to the interpretation of group data and trial design, than to prognosis and clinical management at the individual patient level,.

The role of osteopontin splicing isoforms, the main ones being OPN-a (full-length), OPN-b (Δ-exon-6), and OPN-c (Δ-exon-5), have been extensively characterized in cancer research [52]. These splicing isoforms are observed in humans (and larger mammals such as dogs), but not in mice. Exons 5 and 6 contain transglutamination sites that allow the aggregation of osteopontin homopolymers, which increases their chemotactic, and therefore pro-inflammatory ability. A study of osteopontin isoforms in human and canine muscle [32] identified OPN-a, the full-length and transglutamination-prone isoform, as the most abundantly expressed in dystrophic muscle and mechanically loaded muscles *in vivo*. *In vitro* experiments showed that OPN-a is also the most active isoform in inducing the secretion of several pro-inflammatory cytokines, when administered to human macrophage and myoblast cultures. It is a reasonable assumption, based on predicted molecular weight of spliced exons, that the 55 kDa band identified by immunoblot corresponds to OPNa, while the 50 kDa band corresponds to OPN-b/c. If this assumption were correct, in an attempt to link these findings with those relative to OPN expression studies in deflazacort-treated myoblasts, as summarized above [51], one could speculate that during damage repair in muscle, OPN-a secreted by myoblasts (55 kDa band) is initially pro-inflammatory and chemotactic for neutrophils, while subsequently, fusing myotubes shift to OPN-b/c isoforms (50 kDa band) that are less inflammogenic and modulate regeneration. Further experiments are needed to test this model more thoroughly.

Osteopontin may modify some qualitative aspects of muscle regeneration, other than its speed or efficiency. It is established, since the earliest observations of human dystrophinopathy, as well as of some dystrophin-deficient animal models, such as canine models, that some muscles develop true fiber hypertrophy as a response to dystrophin deficiency [53], while others undergo atrophy and wasting since the early stages of disease. The most potent negative modulator of muscle size, myostatin, presents an expression pattern after muscle damage that is specular to OPN: a brisk downregulation of myostatin, diametrically opposite the upregulation of osteopontin, followed by a gradual return to the baseline levels of both. This observation generated the hypothesis that osteopontin and myostatin signaling may be connected. In fact, experimental evidence showed that recombinant mouse osteopontin induced the phosphorylation of AKT1 and FoxO1, and subsequently a decrease in myostatin, in murine myoblast cultures [54], and that second messengers downstream of osteopontin-integrin interaction at the cell surface mediated this effect. These findings probably explain the mechanistic basis of increased muscle mass in healthy women carrying the G genotype at rs298357094, whose *SPP1* promoter is probably activated by estrogens [49,50]. Furthermore, they add one more layer of complexity to the role of osteopontin in muscle remodeling in dystrophin deficiency—it has to be reminded that muscle hypertrophy may be beneficial in dystrophic patients and animals, insofar as it increases strength, but it may be detrimental when it exacerbates agonist-antagonist imbalance and joint contractures.

In summary, osteopontin is a multifaceted modifier, which acts on both the “acute inflammation” features following early muscle damage in dystrophinopathy, and the efficiency of the regenerative process that follows. The acute inflammatory effect is probably mediated by the chemotaxis of neutrophils and lymphocytes, while the chronic pro-fibrotic effect by modulating myoblast proliferation and macrophage polarization (Figure 1). All of this happens under the complex influences of endogenous steroid hormones and exogenous therapeutic corticosteroids, so that *SPP1* genotype may be considered as a pharmacodynamic biomarker of the GC treatment response in DMD.

This is probably a good point to briefly mention an early study of genetic modifiers of DMD. In 2006, in a subgroup of 48 GC-treated patients from the Padova DMD cohort, three patients were identified who carried a missense N363S SNP (rs56149945) in the *NR3C1* gene (a.k.a. *GRL*, MIM *138040) encoding the glucocorticoid receptor [55]. These three patients incurred into later LoA when compared to the rest of the population. However, the low MAF of this SNP hindered a statistically significant comparison, and the association was not validated in later studies (negative in the CINRG cohort, unpublished data); therefore, it remains to be considered putative.

## 4. *LTBP4* (Latent Transforming Growth Factor β Binding Protein 4)

The path that lead to the identification of *LTBP4* (MIM *604710) as a modifier of DMD is similar to that followed in the case of *SPP1*, insomuch as *LTBP4* was identified as a candidate modifier after a first step that applied a genome-wide technique. However, this was not a gene expression array in human muscle samples, but a genomic linkage study in an animal model [56], namely a γ-sarcoglycan deficient mouse that was derived from the interbreeding of two different parental backgrounds, giving rise to variably severe dystrophic phenotypes. One of the strengths of this study was the careful phenotyping of the animals, which included measures of muscle fibrosis and membrane permeability. A strong association signal with both phenotypes was observed on murine chromosome 7, and fine-mapped to a 36 bp insertion/deletion (indel) polymorphism within the murine *Ltbp4* coding sequence (*Ltbp4^+36^*/*Ltbp4^Δ36^*). The LTBP4 protein is homologous between mice and humans, and it is part of a superfamily of TGFβ binding proteins, including other LTBPs and fibrillins [57,58]. LTBP4 is preferentially expressed into smooth and skeletal muscle, and is co-secreted into the extracellular matrix together with TGFβ, in the form of a large LTBP-TGFβ “latent” complex. This complex adheres to the matrix via the amino-terminus of the LTBP, and it releases TGFβ upon proteolysis by a range of proteases [59,60,61]. The 36 bp indel associated with muscle fibrosis and membrane permeability in dystrophic mice lies within the proline-rich domain of LTBP4, which is the target of proteolysis, and the insertion of the 12 aminoacids encoded by the 36 bp involved in the indel confer resistance to proteolysis [56], reducing the availability of free TGFβ for signaling to its receptors at the plasma membrane. A reduction in TGFβ signaling, in turn, is in keeping with a parallel reduction of fibrosis and membrane permeability, as observed in *Ltbp4^+36^* animals.

Common polymorphisms are very seldom conserved across species; therefore, it is not surprising that the 36 bp indel in *LTBP4* does not exist in humans. However, a haplotype of four missense SNPs along the coding sequence of *LTBP4* is observed in human populations: rs2303729 (V194I), rs1131620 (T787A), rs1051303 (T820A), and rs10880 (T1140M). The two major haplotypes, which account for more than 80% of alleles in most human populations, are VTTT and IAAM. Given the shared pathological mechanism between the loss of dystrophin and its associated proteins, such as sarcoglycans, this variant represented an attractive candidate modifier of human DMD, which was tested in the UDP severe dystrophinopathy cohort (*n* = 254) [45]. In this cohort, the IAAM haplotype was associated with a delay in LoA of around 1.5 to two years, with a recessive inheritance model, and also a strong statistical significance after accounting for the covariates of GC treatment (at least six months before LoA) and predicted truncating vs. non-truncating *DMD* mutations. In fact, the UDP cohort was not strictly defined as a DMD cohort (although DMD was vastly preponderant), but as a “severe dystrophinopathy” cohort, including patients who had lost ambulation before the age of 20, with no “censored” participants who were still ambulatory when last evaluated. Each of the four SNPs in the haplotype were independently associated with later LoA, in a recessive model, despite the strongest modifier effect being associated to the full IAAM haplotype. The most significant single-SNP association was identified at rs10880 (T1140M), situated near the cysteine-rich domain that is implicated in TGFβ binding [59]. The LoA association studies were accompanied by *in vitro* experiments on fibroblast cultures with homozygote VTTT, heterozygote, and homozygote IAAM genotypes. In confluent conditions, with equal levels of LTBP4 expression, the IAAM allele was additively associated with reduced TGFβ signaling, as assayed by immunoblot of the second messengers downstream of the TGFβ receptor, the SMAD proteins. Increased sequestration of TGFβ in the latent complex by IAAM isoprotein, which is probably resistant to proteolysis and/or binds TGFβ, with increased avidity [62], is the basis of the protective effect of this genotype in DMD.

Independent validation studies of the *LTBP4* modifier effect were not late in being published. Using the same recessive model, the Bio-NMD research consortium later confirmed LoA associated with the homozygote IAAM haplotype in their DMD cohort (*n* = 265 genotyped patients), with a haplotype effect being observed in both GC-treated and untreated participants [44]. However, in the CINRG-DNHS cohort, the haplotype effect was not significant in the whole cohort [46]. The CINRG-DNHS is a global multi-ethnic cohort with a slight majority of European-American participants, but also a considerable amount of participants of other ethnic backgrounds, such as European, African-American, Asian-American, Hispanic, and European-Australian (South Asian participants had also been enrolled in the DNHS, but their DNA was not available because of regulatory issues). Therefore, it was hypothesized that a lack of validation of the LTBP4 association may be due to population stratification bias, i.e., bias deriving from the association of different allele frequencies and haplotypic structures at the *LTBP4* locus, with other unknown environmental and genetic modifiers selectively acting in certain ethnic subgroups. In fact, it was clear that some racial/ethnic backgrounds were associated with earlier LoA in the DNHS cohort, e.g., Hispanic. A principal component cut-off was applied to a multidimensional scaling analysis (MDS) of genome-wide SNP markers, that were available from a concurrent modifier discovery study conducted on the same population, in order to objectively select a more homogeneous population (see the section on *CD40*). The selected subpopulation, with few exceptions, self-identified as European/European-American. In this smaller cohort (*n* = 118), which was more homogeneous in terms of ancestry, the recessive effect of the T genotype at rs10880 (corresponding to the “M” in the IAAM haplotype) was significantly associated with later LoA; in particular, in homozygote GC-treated participants, median LoA was delayed to 16.0 years, as compared to 12.9 in the remaining cohort. This finding highlighted the importance of controlling and accounting for population stratification bias in genetic modifier research. Later studies showed that the haplotypic structure at the *LTBP4* locus differs between populations of different ancestries, with strong linkage disequilibrium (LD) and clear-cut distribution between the major VTTT and IAAM haplotypes in European-Americans, vs. comparatively disrupted LD and more frequent minor haplotypes in African-Americans [62]. It could be that the modifier effect is stronger when the variants are co-inherited within the VTTT/IAAM diplotype, or that other coding or regulatory variants are at play with different degrees of LD. In fact, recent evidence points in the latter direction, as explained below.

Recently, the UDP group published the result of a GWAS of the age at LoA in the same cohort (UDP severe dystrophinopathy), in which the *LTBP4* association was originally described. Despite that the size of the cohort (*n* = 253) may be considered to be very small for GWAS standards, the authors were able to identify and functionally characterize two modifier loci with genome-wide significance [63], with a recessive inheritance model. While the adoption of a fully recessive inheritance model is uncommon in GWASs of quantitative traits, the authors reasoned that as the previously identified modifiers (*SPP1* and *LTBP4*) show dominant and recessive risk patterns, including these models in their analysis would increase power. One of the identified loci was tagged by the SNP rs710160, situated around 12 kb upstream of the *LTBP4* promoter. This upstream position is strongly suggestive of a regulatory role and, in fact, data from public expression quantitative trait locus (eQTL) databases confirmed that the minor C allele at rs710160 is associated with reduced *LTBP4* transcriptional activity. When the rs710160 genotypes were phased with the VTTT/IAAM haplotype, it became evident that the minor C allele “splitted” the IAAM allele, the major haplotypes being T-VTTT (49%), C-IAAM (17%), and T-IAAM (13%). C-IAAM emerged as the haplotype that is associated with both the latest LoA in the UDP cohort, and the lowest *LTBP4* expression in re-analyzed public eQTL data, which derives from cell cultures from populations of various ancestral backgrounds. The authors proposed a model where a low expression of a highly proteolysis-resistant and TGFβ-avid LTBP4 isoform—predicted by the homozygous C-IAAM allele—leads to the least pro-fibrotic signaling and the mildest phenotype (Figure 1). Preclinical data also supports this model. In fact, the over-expression at the mRNA level of the murine *Ltbp4* gene in *mdx* had led to an amelioration of the dystrophic process, which was probably because of increased retainment of TGFβ within the latent complex [64], while the expression of the human “risk” VTTT allele in *mdx* lead to an exacerbation of fibrosis and an aggravation of the phenotype [65]. The human LTBP4 protein has a shorter hinge region, which makes the latent complex more readily attacked by proteases. So to speak, the murine 36 bp deletion makes LTBP4 “more like” the human protein, by shortening the hinge; while the human IAAM isoprotein makes LTBP4 “more like” the murine protein, by stabilizing the complex. We may even suppose that differences in LTBP4 and TGFβ signaling might be one of several reasons for the alleviated phenotype of dystrophin deficiency in mice, when compared to larger mammals.

A last consideration regarding the identification of the regulatory rs710160 SNP by GWAS is that its LD with the VTTT/IAAM haplotype provides a good explanation of its varying effect between populations of different ancestries. A simple measurement of pair-wise LD between rs710160 and rs10880 in the CEU (European-American) population from the 1000 genome project (https://ldlink.nci.nih.gov, last accessed 5 February 2019) provides a value of *R*^2^ of 0.33 and D’ of 0.61, i.e., a strong LD, while the same values show that *R*^2^ = 0.01, D’ = 0.10 in MXL (Mexicans from Los Angeles), and *R*^2^ = 0.00 and D’ of 0.09 in ASW (African-Americans from South-West USA), i.e., linkage equilibrium in non-European populations. Therefore, the preferential effect of the IAAM haplotype in the CINRG-DNHS subgroup of Caucasian ancestry [46] may have been due to higher LD with the regulatory variant rs710160.

## 5. *CD40*, a.k.a. *TNFRSF5* (Tumor Necrosis Factor Receptor SuperFamily Member 5)

About two years before the UDP group published the GWAS cited above, another attempt at genome-wide discovery of SNPs implicated in modifying the DMD phenotype was made by the CINRG consortium [66]. The peculiarity of this study was the adoption of the Exome Chip, a genotyping chip that is focused on variants that are situated within gene-coding regions and their immediate vicinities. The Exome Chip was originally designed for the purpose of validating associations with moderately rare variants that were identified by exome sequencing (https://genome.sph.umich.edu/wiki/Exome_Chip_Design). To this end, the chip includes a large quantity of low-frequency, coding variants (MAF < 0.05). However, it was also enriched with several thousands of common (MAF > 0.05) SNPs, which are also situated in coding or regulatory regions, with known effects on protein (e.g., missense or nonsense) or RNA expression, or known to be associated with phenotypic effects from previous GWASs, or with other relevant biological/clinical annotations. This selection of “functional” common SNPs is relatively small (~27,000) when compared to the hundreds of thousands of common SNPs that are needed to homogeneously tag the genome in “classic” GWASs. Therefore, it was hypothesized that using the Exome Chip would have led to a lower false discovery rate, due to the smaller number of parallel statistical tests involved. Such an approach was necessary, in view of the very small size of the CINRG-DNHS cohort for GWAS standards. In fact, the Exome Chip genotypes were obtained in only 175 participants with sufficient DNA sample quantity and quality. Moreover, in order to prevent a severe bias from population stratification, as explained above for *LTBP4*, association studies were limited to 109 unrelated participants of European ancestry, after the exclusion of MDS outliers. Association with age at LoA was studied in 27,025 SNPs with MAF > 0.05, with GC treatment for at least one-year before LoA as a binary covariate in a Cox regression model. No SNPs exceeded the Bonferroni-corrected threshold of “exome-wide” significance; therefore, the authors applied a hypothesis-driven selection of SNPs within genes that were implicated in fibrosis and inflammation, defined as belonging to the NF-κB and TGFβ pathways according to Gene Ontology annotations (438 genes). When applying an accordingly redefined Bonferroni threshold to the association p-values, the two SNPs rs6074022 and rs4810485, which corresponded to a haplotype around the promoter and 5’ region of the *CD40* gene, stood out as significant, with the minor allele being associated with a 2.8-year earlier LoA in a dominant model (*p* = 1 × 10^–4^), and a 2.1 GC-treatment corrected hazard ratio in an additive, “per-copy” model (*p* = 3.4 × 10^–5^). Genotyping rs1883832, a third SNP in 100% LD with the two mentioned above, validated the association, in a multicenter cohort comprised of: the remaining CINRG-DNHS participants of self-identified European ancestry who had not been genotyped with the Exome Chip; the UDP severe dystrophinopathy cohort [45,63]; the Bio-NMD DMD cohort [44]; and, the Padova DMD cohort [27] (total *n* = 660). In this validation cohort, one-year earlier LoA was observed in association with the minor T allele (dominant model *p* = 0.002, additive *p* = 0.02).

*CD40* (MIM *109535) encodes TNFRSF5, which is a crucial co-stimulatory protein expressed on antigen-presenting cells (APCs). Its interaction with its ligand, expressed on the surface of T-cells, is necessary for their activation through T-cell receptor—major histocompatibility complex interaction. rs1883832, as expected for a common SNP selected for the Exome Chip, is rich in functional annotations. It is situated in the 5’ untranslated region of *CD40*, adjacent to the translation start site, and within a Kozak sequence relevant for ribosome binding. There is a long list of associations with several autoimmune diseases, including, among others, Graves disease [67], multiple sclerosis [68], and Kawasaki disease [69]. Notably, the minor T allele was found to confer an increased risk of some of these autoimmune conditions, but to be protective against others, pointing to complex immune network interactions and disease- and tissue-specific pathogenetic processes.

The exact role of *CD40* in muscle dystropathology is poorly understood. It would be a circular argument to just state that T-cell activation triggers the NF-κB pathway: according to the study design explained above, the association signal positioned around *CD40* was singled out *because* it came from a gene in this pathway. However, it can be said that the transition from innate immunity, which is stimulated in the presence of necrotic muscle fibers by the liberation of damage-associated molecular patterns, to cell-mediated immunity, which in turn orchestrates the recruitment of M1/M2 macrophages and the onset of regeneration, is crucial in determining the success of tissue repair, or, on the contrary, its failure, which results in end-stage fibrosis [70]. Furthermore, there is ample evidence from dystrophic animal models and patient tissues that T-cell depletion modulates both fibrosis [71,72,73,74,75,76] and GC treatment response [77].

The delineation of a mechanistic model, linking the modifier SNP genotype to the phenotype, is complex for *CD40* as it was for *SPP1* and (maybe to a lesser extent) *LTBP4*. rs1883832 is in perfect LD with an upstream promoter SNP (rs6074022, initially identified by Exome Chip), whose minor allele reduces *CD40* transcriptional activity; moreover, the minor haplotype at this locus has been associated with the increased expression of a Δ-exon-6 secreted isoform, which may act as a decoy receptor and prevent cell-cell interactions between the APCs and T cells [68]. Altogether, it appears that the minor allele, associated with earlier LoA, steers the system towards reduced *CD40* activity and subsequent reduced T cell activation. This is quite counter-intuitive, as most studies in animal models describe a reduction of fibrosis, when the T cells are depleted [73]. However, the contrasting effects of rs1883832 in different immune diseases remind us that the network of potential compensatory events in the immune pathology of dystrophin deficiency is vast, and the widespread administration of GCs in the DMD population further complicates the picture. Preliminary studies of its expression in DMD muscle biopsies were attached to the report of *CD40* as a DMD modifier [66] (at diagnosis, priori to GC treatment), which surprisingly showed increased levels of *CD40* transcript, assessed by RT-PCR, in association with the T allele (*n* = 16, *p* = 0.005), but expectedly, reduced levels of CD40 protein with the same allele (*n* = 6, *p* = n.s.). Further studies are needed to clarify the exact events connecting *CD40* genotype to modified DMD pathology (Figure 1).

## 6. *ACTN3* (Actinin-3)

Sarcomeric α-actinins are major components of the sarcomeric Z-line, where they bind and cross-link the sarcomeric actin filaments. While α-actinin-2 is ubiquitous in all muscle fibers, α-actinin-3 is selectively expressed in fast, glycolitic fibers [78]. A common null polymorphism (R577X, rs1815739) in the *ACTN3* gene (MIM *102574), which codes α-actinin-3, determines the complete absence of this protein in 16% of individuals (18% of those of European ancestry) [79]. This polymorphism represents an interesting example of a “human knockout” of an important muscle protein [80], and it has emerged as a major association in the genetics of exercise. In fact, the null allele is detrimental to the sprint and power performance both in normal individuals [81] and in athletes [79,82]. Conversely, endurance performance has been reported to be improved in athletes carrying the same allele [82,83], although with some doubts [84]. The ablation of the homologous *Actn3* murine gene recapitulated the human “577X” phenotype, with reduced muscle mass and strength, but improved response to resistance training [85,86].

A common polymorphism, with such a large effect on muscle function in health, represented an excellent candidate as a DMD modifier. In fact, rs1815739 had been included in the multi-candidate gene study that had led to the identification of *SPP1* as a modifier [27], and reported as being associated to grip strength in the CINRG-DNHS cohort; however, the association could not be significantly cross-validated with the Padova cohort at the time, and it remained unconfirmed. More recently, new data in the enhanced CINRG-DNHS cohort [87] strengthened the association, and extended it to quantitative muscle testing in several districts (elbow and knee extensors, knee flexors) and, importantly, to 10 m walk/run velocity, a widely used outcome measure in DMD clinical trials. As expected, the null alleles were associated with reduced power and speed. The effect on LoA, which is the principal phenotype used for the evaluation of genetic modifiers, was harder to explain, as heterozygous (RX) participants suffered earlier LoA with a median age of 11.7 years (95% CI 11.0–13.0), as opposed to 12.5 (11.1–14.0) in RR and 13.0 (11.9–14.0) in XX. Finding a rationale to this “genotypic” model, with similar outcomes in discordant homozygotes, is challenging. A re-analysis of the Padova cohort confirmed earlier LoA in both RX and XX patients; and a meta-analysis of 297 participants of European ancestry in the CINRG and Padova cohorts confirmed a genotypic model with earlier LoA in heterozygotes.

The same paper that reported on associations between *ACTN3* genotypes and functional muscle phenotypes in the CINRG-DNHS [87] also included an extensive phenotypic and histological characterization of a double knock-out (dKO) mouse that was obtained by crossing α-actinin-3 deficient mice with *mdx*. dKO mice, as expected, showed reduced muscle size and strength (as well as size-adjusted strength) when compared to *mdx* controls, but also a reduction in muscle fiber necrosis, increased regeneration, and less weakness induced by an eccentric exercise challenge. The authors identified the reason for this protective effect of α-actinin-3 deficiency in an activation of calcineurin signaling, inducing a shift in the balance of muscle metabolism from glycolytic towards oxidative; and the oxidative fibers have long been known to be less affected by degeneration that is caused by dystrophin deficiency [88]. LoA in human patients is a more complex phenotype than strength, power, or histological measurements, as it entails the interaction of several compensatory factors, such as the relative involvement of different muscle groups and joint contractures. It may be that partial α-actinin-3 deficiency in rs1815739 RX heterozygote DMD patients is enough to cause a reduction in muscle power, but not enough to activate the compensatory pathways that are needed for a protective shift towards oxidative metabolism. In fact, a dose-dependent, “haploinsufficiency” model of α-actinin-3 deficiency in muscle had been previously proposed in heterozygous *Actn3* (+/-) mice [87].

## 7. *THBS1* (Thrombospondin-1)

The GWAS that was conducted by the UDP consortium in their severe dystrophinopathy cohort [63], in addition to identifying the regulatory SNP upstream of *LTBP4* that was described above, identified a distinct association signal, corresponding to the SNPs rs2725797 and rs2624259, which are in close proximity and strong LD (*R*^2^ = 0.95) at 15q14. This association signal resulted in genome-wide significance using a recessive inheritance model (*p* = 6.6 × 10^–9^), but a suggestive *p*-value of 7.5 × 10^–6^ was also obtained with an additive model. As these SNPs are situated within a large “gene desert”, in order to decipher the biological underpinnings of the association, the authors applied elegant *in silico* techniques, leveraging publicly available haplotype structure and gene expression data. They performed SNP genotype imputation in the genomic region, identifying a strong LD block of SNPs in strong LD (*R*^2^ > 0.8). Subsequently, they were able to demonstrate long-range chromatin interactions between this region and the promoter of the *THBS1* gene (MIM *188060) encoding thrombospondin-1, which is situated about 750 kb telomeric from the association signal. This was possible by mining data from a public database of interactions between gene promoters and genomic regions that were identified by promoter capture Hi-C [89], according to which the *THBS1* promoter specifically interacts with restriction fragments containing the highest-associated SNPs, rs2725797 and rs2624259. These SNPs flank a strong enhancer site, which is likely mediated by a consensus site for CCCTC-binding factor (CTCF). Furthermore, mapping of DNAse I hypersensitivity sites confirmed that, in the vast majority of examined cell types, the corresponding DNA stretch takes a conformation of open chromatin, which is suggestive of a cis-regulatory element. Finally, eQTL data from the GTEx Project list the majority of SNPs in strong LD with rs2725797 and rs2624259 among the top associations with *THBS1* expression, again across multiple tissues. Collectively, the evidence that these SNPs tag a crucial regulatory region of *THBS1* is very strong, despite the considerable genomic distance. The minor allele at rs2725797, associated to reduced *THBS1* expression, appeared to be protective against DMD progression (i.e., later LoA). Thrombospondin is a major activator of TGFβ *in vivo* [90], by exposing its active form through proteolysis of the latent complex (Figure 1). Therefore, it is straightforward to include this novel association into a larger model, where TGFβ activation is a central checkpoint of muscle tissue repair in dystrophin deficiency, regulating the balance between successful regeneration, or its failure, and the onset of fibrosis.

## 8. Alternative (Non-Skeletal Muscle) Phenotypes

Most studies of the association between DMD phenotype variability and modifier polymorphisms have focused on the LoA phenotype. This is justified by several facts, as mentioned above: LoA is a cardinal event in the disease natural history; it is easily measurable and recollectable with precision even after several years; it correlates well with other meaningful milestones; and, maybe most of all, it provides a good estimate of overall disease severity, representing the culmination of life-long pathological processes and the result of several compensatory mechanisms —more so than punctual measures of muscle strength and function, such as motor scales, manual or quantitative strength measurements, or timed tests, which are more volatile and prone to modification, due to random contingent factors. However ambulation, and even motor function itself, may not always be considered to be the most important phenotypic aspect of DMD. Respiratory and cardiac insufficiency, for instance, more strongly affect life expectancy than skeletal muscle weakness, and they are the leading causes of death in DMD.

The onset of respiratory insufficiency is at least as variable as skeletal muscle weakness in DMD, as there is considerable variation in the age at which patients reach relevant milestones, such as forced vital capacity (FVC) falling below 50% of the value that is predicted by age and height, or below the absolute value of 1 L. These spirometric findings strongly predict the appearance of night-time hypoventilation, leading to hypercapnia, and subsequent dependence from nocturnal non-invasive ventilation [91]. Respiratory insufficiency is usually more severe in DMD patients with weaker skeletal muscles and more severe functional impairment [10], so that all of the modifiers of skeletal muscle strength may be considered as putative modifiers of respiratory insufficiency. In fact, the same pathological processes of secondary inflammation, attempted regeneration, and end-stage fibrosis occurs in the diaphragm as in other skeletal muscles, although with possible differences, as suggested by expression arrays in animal models [92,93]. To this day, formal association studies of human SNPs with respiratory outcomes in DMD are lacking, but these will no doubt emerge as natural history cohorts and are more extensively phenotyped [94,95] and genotyped in the near future.

The progressive reduction in the contractility and increase in volume of the left ventricle of the heart, leading to DCM, is less obviously correlated with the severity of skeletal muscle involvement, to the point that some authors have even argued that weaker patients may be protected by exertion-induced stress on the myocardium [96]. Usually, DCM presents with moderate abnormalities that are identified by echocardiography in the early non-ambulatory phase, and progresses to severe heart failure in late non-ambulatory stages. However, a continuous spectrum is observed between DMD patients who retain a normal heart function throughout adult life, despite the early loss of motor function, and conversely those who develop life-threatening heart failure in early non-ambulatory phases, while upper limb function is still preserved. The identification of genetic factors that influence DCM onset would have great prognostic relevance, and may guide prophylactic drug treatment. Importantly, it should be pointed out that, while a fairly strong genotype-phenotype correlation between the position of *DMD* mutations and the risk of DCM has been described in BMD, proximal deletions in the N-terminal actin-binding domain being especially involved [97], such a correlation has never been found in DMD. Therefore, a potential strong effect of “trans-active” modifiers is postulated.

The Padova group, in collaboration with several other Centers of the Italian DMD Network, genotyped a cohort of 178 DMD patients with available retrospective echocardiographic data, and performed a time-to-event analysis of age at onset of DCM [13]. This is a challenging phenotype to study, as the onset of DCM is almost always asymptomatic. In this study, DCM onset was defined as the age at the first appearance of a left ventricular ejection fraction (EF) below 50% and/or a left ventricular end diastolic volume (EDV) above 70 mL/m^2^ of body surface. In order to be able to correctly identify the age at onset, the authors only included patients who had a normal echocardiogram preceding the first abnormal one by twelve months or less. The findings in the whole population were confirmative of the known natural history of DMD-related DCM [98], as the median age at DCM onset was 20 years. There was no clear effect of GCs in delaying DCM onset, and in fact, the literature on this point is discordant [99,100,101,102,103]. The cohort was genotyped for the genetic modifiers of skeletal muscle function in DMD known until then (2015), i.e., *SPP1* rs28357094 and the *LTBP4* VTTT/IAAM haplotype. The G allele at rs28357094, which had been described as being detrimental in skeletal muscle, was not associated to earlier DCM, but if anything to a non-statistically-significant trend towards *later* DCM,. For the IAAM haplotype (protective in skeletal muscle), on the other hand, the data did also suggest a protective role against the onset of cardiac abnormalities, which was statistically significant in the subgroup of GC-treated participants. TGFβ is a well-known driver of dilative ventricular remodeling in dystrophic and non-dystrophic cardiomyopathies, as it stimulates myocardial fibrosis [104,105], and angiotensin-converting enzyme inhibitors (ACEi) are effective against cardiac remodeling, because they block downstream TGFβ activation [106]. Therefore, the putative protective role of the IAAM haplotype, which, as extensively explained above, stabilizes the TGFβ within the latent complex, has strong biological justification. However, from a genetic association standpoint, the paper that is summarized here can only be considered to be exploratory because of several limitations: the relatively small sample size, the lack of a precise analysis of the effect of cardiological treatments, such as ACEi and beta-blockers (due to the retrospective nature of the study), and the difficulty to relate the echocardiographic onset of DCM to the actual development of symptomatic and life-threatening heart failure in the following years. Therefore, further studies in larger populations, possibly with longitudinal designs, are warranted in order to decipher the genetic bases of DCM variability in DMD. The main challenge to longitudinal studies will be represented by the long periods of observation that are needed to develop DCM over time.

## 9. Conclusions

Despite the several genetic associations with different sub-phenotypes of DMD that are presented in this review (Table 1), we feel that our knowledge in this field is still initial. Studies have heavily relied on hypotheses (candidate genes) in order to identify and validate putative modifiers. Correspondingly, the most relevant modifiers that have been identified so far are implicated in pathways that were already well established as drivers of secondary inflammation, fibrosis, and failure of regeneration in dystrophin deficiency. Alternatively, they were already known to modify the functional properties of healthy muscle. The identification of modifier SNPs in “usual suspect” genes is very relevant for patient stratification and potential prognostic uses, but it contributes relatively little to the identification of novel therapeutic targets.

An unbiased, hypothesis-free approach, such as GWAS, would have the power to identify “unsuspected” associations, with the potential of discovering novel therapeutic targets. While dystrophin restoration certainly appears to be the main avenue for treating DMD, there are many hurdles and limitations along this path. Furthermore, it is suggested by some animal models [107] and human cases [108] that, in some unclear way, skeletal muscle may find a way to tolerate dystrophin deficiency much better than normally observed. Therefore, it is possible that targeting potent, yet undiscovered DMD genetic modifers may significantly benefit patients. Initial attempts to identify modifiers at the genomic scale [63,66] were limited due to sample size and/or methodological issues. Large-scale national and international collaborations will be paramount, as GWAS standards require sample sizes that are usually inaccessible in rare diseases.

As a last consideration, DMD may be considered as a paradigm for modifier studies in neuromuscular diseases, and rare Mendelian diseases in general, as it is relatively common (among rare diseases), intensely studied at the phenotypic and pathological levels, and relatively homogeneous as far as the causative defect is concerned. A great challenge for the future will be represented by the characterization of modifiers in rare diseases at large. Genetic diagnoses are increasingly reached by next generation sequencing approaches, and diagnostic samples are increasingly studied with transcriptomic and proteomic methods which provide a wealth of information regarding the genetic background of the proband and the molecular pathways involved in the pathogenesis. Therefore, we propose a vision in which the globality of this information is taken advantage of, for the construction of a personalized precision medicine for rare disease patients.

## Figures and Tables

**Table 1:** DMD modifier loci, corresponding SNPs, and their associations with DMD phenotypes.

Locus	Protein Product	rs#	Alleles	Chr Position (GRCh38.p12)	Inheritance Model	SNP Effect	Minor Allele Effect on Protein Product	Minor Allele Effect on DMD Severity	Studied DMD Sub-Phenotypes	Outcome Measures	Positive Association Studies (*n* of DMD Participants)		Negative Association Studies (*n* of DMD Participants)		Ref.
											Discovery	Validation	Discovery	Validation	
*SPP1*	Secreted PhosphoProtein 1, also known as osteopontin	rs28357094	T>G	4:87975645	Dominant	Transcriptional (promoter)	Reduced expression, increased steroid responsiveness	Detrimental	Skeletal muscle strength	MRC strength	80	-	-	-	43
										Grip strength	156	-	-	-	27
										NSAA (1-year change)	80	-	-	-	
										6MWT (1-year change)	80	-	-	-	
										LoA	106	279	-	254; 336	27, 44, 45, 46
									Dilated cardiomyopathy	Onset (age at LVEF < 50% or LVEDV > 70 mL/m^2^)	-	-	178	-	13
*LTBP4*	Latent Transforming growth factor β Binding Protein 4	rs2303729, rs1131620, rs1051303, rs10880	G>A, A>G, A>G, C>T	19:40605163, 19:40611963, 19:40612150, 19:40622404	Recessive	Coding haplotype (VTTT/IAAM)	Resistance to proteolysis, increased TGF-β binding avidity	Protective	Skeletal muscle strength	LoA	254	274; 265	-	137	13, 44, 45, 46
									Dilated cardiomyopathy	Onset (age at LVEF < 50% or LVEDV > 70 mL/m^2^)	178	-	-	-	13
		rs710160	T>C	19:40581585	Recessive	Upstream regulatory	Reduced expression	Protective	Skeletal muscle strength	LoA	253	-	-	-	63
*CD40*	Tumor Necrosis Factor Receptor SuperFamily member 5 (TNFRSF5)	rs1883832	C>T	20:46118343	Additive/ dominant	5’-UTR (Kozak sequence)	Reduced expression	Detrimental	Skeletal muscle strength	LoA	109	660	-	-	66
*THBS1*	Thrombospondin-1	rs2725797	G>A	15:38817032	Recessive	Long-range regulator	Reduced expression	Protective	Skeletal muscle strength	LoA	253	-	-	-	63
*ACTN3*	α-actinin-3	rs1815739	C>T	11:66560624	Additive/ genotypic	Nonsense	Complete defect	Detrimental (worse in heterozygotes?)	Skeletal muscle strength	Grip strength	59	-	-	-	87
								Detrimental (worse in heterozygotes?)		QMT strength	61	-	-	-	
								Detrimental (worse in heterozygotes?)		10 m run/walk speed	61	-	-	-	
								Detrimental (worse in heterozygotes?)		LoA	266	102	-	-	

DMD: Duchenne muscular dystrophy. SNP: single nucleotide polymorphisms. Chr: chromosome. MRC: Medical Research Council manual muscle testing score. NSAA: North Star Ambulatory Assessment. 6MWT: 6 Minute Walk Test. LoA: loss of independent ambulation. LVEF: left ventricle ejection fraction. LVEDV: left ventricle end diastolic volume. 5’-UTR: 5’ untranslated region. QMT: quantitative muscle testing.
